# The Undoped Polycrystalline Diamond Film—Electrical Transport Properties

**DOI:** 10.3390/s21186113

**Published:** 2021-09-12

**Authors:** Szymon Łoś, Kazimierz Fabisiak, Kazimierz Paprocki, Mirosław Szybowicz, Anna Dychalska

**Affiliations:** 1 Institute of Mathematics and Physics, Bydgoszcz University of Science and Technology, Profesora Sylwestra Kaliskiego 7, 85-796 Bydgoszcz, Poland; 2 Institute of Physics, Kazimierz Wielki University, Jana Karola Chodkiewicza 3, 85-064 Bydgoszcz, Poland; paprocki@ukw.edu.pl; 3 Faculty of Materials Engineering and Technical Physics, Institute of Materials Research and Quantum Engineering, Poznań University of Technology, Piotrowo 3, 61-138 Poznań, Poland; miroslaw.szybowicz@put.poznan.pl (M.S.); anna.dychalska@put.poznan.pl (A.D.)

**Keywords:** hydrogenation impact, diamond films, XRD, dc-conductivity, Raman spectroscopy, HF CVD

## Abstract

The polycrystalline diamonds were synthesized on n-type single crystalline Si wafer by Hot Filament CVD method. The structural properties of the obtained diamond films were checked by X-ray diffraction and Raman spectroscopy. The conductivity of n-Si/p-diamond, sandwiched between two electrodes, was measured in the temperature range of 90–300 K in a closed cycle cryostat under vacuum. In the temperature range of (200–300 K), the experimental data of the conductivity were used to obtain the activation energies *E*${}_{a}$ which comes out to be in the range of 60–228 meV. In the low temperature region i.e., below 200 K, the conductivity increases very slowly with temperature, which indicates that the conduction occurs via Mott variable range hopping in the localized states near Fermi level. The densities of localized states in diamond films were calculated using Mott’s model and were found to be in the range of $9\times 10^{13}$ to $5\times 10^{14}\;{\mathrm{eV}}^{-1}{\mathrm{cm}}^{-3}$ depending on the diamond’s surface hydrogenation level. The Mott’s model allowed estimating primal parameters like average hopping range and hopping energy. It has been shown that the surface hydrogenation may play a crucial role in tuning transport properties.

## 1. Introduction

Due to diamond’s attractive properties, including wideband gap, high breakdown voltage, small dielectric constant, and excellent radiation hardness, it is recognized as a future material suitable for microelectronics devices that can operate at high temperatures and in chemically harsh environments [1]. Contemporary trends of electronics development make a request of sensing device growth, especially cheap portable gas sensors [2,3,4,5]. Due to the rare feature of the negative value of electrons affinity of polycrystalline diamond layers, they seem to be one of the best such materials [6]. As the interaction between gas molecules and the diamond hydrogen-terminated surface should lead to the change of layer’s resistance [7]. To use diamond layers to realize reliable electronic devices, several problems are still to be solved. In particular, the electrical properties of diamonds, including the role of hydrogen, which seems to be one of the most important, is not fully clarified at present but can be the key parameter in the design of electronic devices. For over 20 years, many efforts have been made to explain the role of hydrogen in the electrical conductivity of hydrogenated diamonds. The first report on this problem appeared in 1989 by Landstrass and Ravi on the electrical conductivity of as-grown CVD diamond layers grown in a hydrogen-rich plasma [8]. They observed that the resistivity of the diamond could increase even by six orders of magnitude when it is annealed at a high temperature, what leads to dehydrogenation. It is generally known that as-grown diamond layers produced by CVD methods are hydrogenated due to the nature of the growth process that takes place in a hydrogen-rich atmosphere, i.e., the ratio is H${}_{2}$/CH${}_{4}$ is about 99/1, and shows p-type surface conductivity [9,10]. In the case of conventional semiconductors like Si and GaAs it is known that hydrogen atoms terminate dangling bonds, passivate shallow and deep levels, and introduce extended defects [11,12]. The interaction of hydrogen with the diamond surface is more complex. It has been shown by Maier F. et al. that the origin of the CVD diamonds’ surface conductivity is related to their hydrogenation [13]. However, the density functional theory calculation has revealed a crucial role of direct bonding of hydrogen to carbon atoms [14]. The created C-H bond has polar character, i.e., it introduces an electric dipole moment and shifts up electrons’ energy by 1.5 eV. The complexity needs further research as, for example, the negative electron affinity of a hydrogenated diamond surface [15]. Polycrystalline diamond/hydrogenated layer consists, at least, of three different components, i.e., diamond microcrystalline, a transitional region between the different orientations of the neighboring crystallizes (hydrogenated sp${}^{2}$-hybridized carbon), and grain boundaries between them. The grain boundaries always contain much incomplete atomic bonding (unsaturated dangling bonds), which can act as a carrier trap center that leads to degradation of device performance. In polycrystalline layers, the intergrain boundary can have a very complex structure, consisting of the thin layer of disordered atoms, which act as an interface between the different orientations of the neighboring crystallizes [16]. In this interface region, due to incomplete bonds between atoms, trapping (immobilization) of carriers occurs, and an energy barrier is created. This barrier reduces the mobility of the carriers when passing from one grain to another one. The mechanisms limiting the electrical carrier’s mobility can also be associated with electron-phonon interaction, scattering by impurities or in grain structural defects [17,18]. The most effective method to characterize the electronic properties of semiconductor materials is to study the mechanisms of electrical transport. In particular, the conductivity measurements (I-V-T) [19] as a function of temperature provide valuable information about the conduction mechanisms and parameters such as charge-carrier density, mobility, and defect ionization energy. J-V-T characteristics for diamond were also analysed by A.M. Rodrigues, et al. and J.C. Madaleno et. al. [20,21]. However, they did not analyze the mechanism of charge current transport, which is one of the goals of this study. Other authors [22,23,24] relate the electrical properties of undoped diamond layers mainly to the sp${}^{2}$ carbon phase content. It should be noted, however, that this phase is generally highly hydrogenated. Changing the surface termination from hydrogen to oxygen increases the resistance by several orders [25,26,27].

In this work, we report a study on the influence of diamond hydrogenation level on the conduction mechanism and electrically active defects in polycrystalline diamond layers prepared by the Hot Filament Chemical Vapor Deposition (HF CVD) technique. The aim of the undertaken research is estimation, based on I-V-T characteristics, of the essential parameters related to the sample’s electronic structure including an activation energy *E*${}_{a}$, a grain boundary barrier (GB) height $\phi_{GB}$, a density of states near Fermi level *N*(*E*${}_{F}$), a hopping energy *W* and a hopping distance *R*.

## 2. Materials and Methods

The polycrystalline un-doped diamond films (DF) were grown at the rate of 0.2 μm/h on (100) oriented monocrystalline n-Si wafers using the HF CVD technique. The deposition was carried out using methane as the carbon-containing gas, diluted in H${}_{2}$. The total gas flow rate was fixed at 100 sccm and the percentage flows for methane and H${}_{2}$ was 3 vol%. The tungsten filament temperature was heated up to 2300 K. For the synthesis of the diamond, layers were chosen three different working gas pressures e.g., 40, 60 and 80 hPa. To enhance diamond nucleation, before the growth process, the substrates were mechanically polished with 1 μm diamond powder in an ultrasonic bath. The growth temperature of the substrate was estimated to be 1100 K. Our experimental set-up for synthesis with more details is presented in [28]. After the deposition process, the diamond surfaces and substrates were metalized by gold evaporation for development of four probes electrode contact. The dc-electrical measurements were performed in a hetero-junction configuration for chosen samples (DF40, DF60, DF80) from a larger set of synthesized diamond films on different parameters of growth process. In our case, we selected samples synthesized at the same working gas composition, changing only its total pressure.

The Raman spectra were recorded at room temperature in backscattering geometry using Renishaw inVia Raman spectrometer. All spectra were recorded in the spectral range of 1000–2000 cm${}^{-1}$ with a 488 nm excitation wavelength generated by tuneable ion argon laser. The spot size and accumulation time were 1 μ$m^{2}$ and 30 s, respectively. The parameters of the obtained Raman spectra were analyzed using Renishaw WiRE 3.1 software.

The diamond film morphology has been studied by Scanning Electron Microscope (SEM), Jeol JSM-6300 operating at a voltage of 25 kV. SEM images were also used to examine grain size and film thickness.

X-ray diffraction patterns were recorded by using DRON-4a, Θ–2Θ XRD diffractometer. A Cu Kα X-ray source was used at 32 kV and 12 mA. The lattice parameters can be extracted from the positions of the diffraction peaks obtained in several directions using the silicon lattice parameter for calibration.

The I-V-T characteristics were performed in Oxford Optistat cryostat in the temperature range 77–300 K using the following instruments: Rigol DG1022A as the power supply generating a rectangular voltage wave with peak to peak amplitude in the range of 4–20 V. The current was registered by Keithley picoammeter of a series 6485 and a potential drop by Fluke 8505A Digital Multimeter. Each measurement temperature was stabilized by Oxford Mercury controller.

## 3. Results and Discussion

### 3.1. SEM Analysis

**Figure 1:**
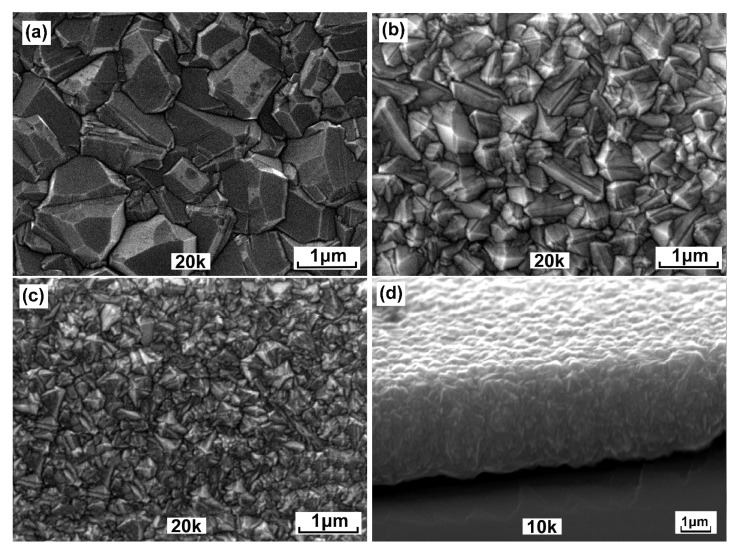
The diamond morphologies synthesized at working gas pressure of (**a**) 40 hPa, (**b**) 60 hPa (**c**) 80 hPa and (**d**) diamond layer cross-section.

In the present studies, three diamond layers were synthesized at different working gas pressures, i.e., 40, 60 and 80 hPa, while other synthesis parameters remained fixed. The morphologies of the obtained diamond films are shown in Figure 1. The obtained thicknesses from their cross-sections were in the range of 3–4 μm. The microcrystal sizes, as observed by SEM, are changing from about 2 μm to below 1 μm depending on the synthesis pressure. Clearly, the grain decreases, and the film becomes microscopically smoother with increasing the pressure of the feed gas. The smoothing of the polycrystalline diamond surface is caused by the extent of secondary nucleation. This also leads to the decrease in the nominal grain size and, in consequence, to the increase in grain boundaries fraction.

### 3.2. X-ray Diffraction Measurements

**Figure 2:**
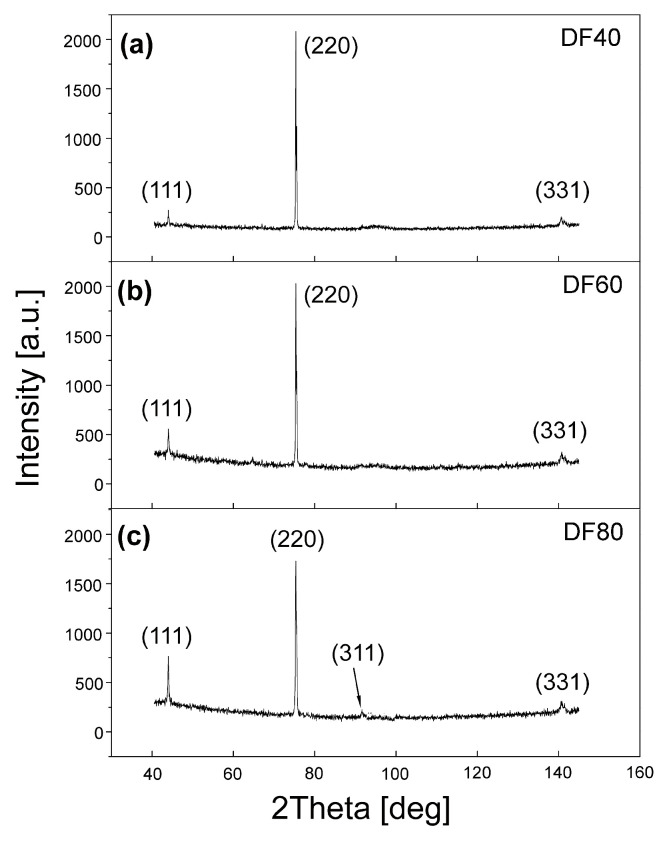
X-ray patterns of the studied CVD diamond films (**a**) DF40, (**b**) DF60 (**c**) DF80.

Figure 2 presents the standard 2Θ X-ray diffraction patterns. They indicate the three characteristic reflections for diamond, i.e., (111), (220) and (331) in the scanned 2Θ range. The most distinct reflex in X-ray diffraction spectra observed for all samples is (220) reflection, which indicates the similar preferential orientation of microcrystallites within the range of variable CVD growth parameters. The average grain sizes (L) were calculated from the half width at half maximum of the Bragg peak, using the Debye-Scherrer formula [29,30,31]. The X-ray diffraction data are summarized in Table 1 together with the results of Raman spectroscopy measurements. It can be seen that the estimated grain sizes change in the same direction as those observed in Figure 1.

### 3.3. Raman Spectroscopy Measurements

**Figure 3:**
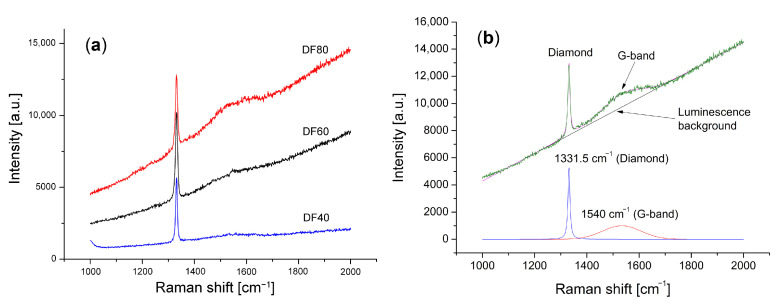
The Raman spectra (**a**) corresponding to the investigated diamond’s films presented in Figure 1, (**b**) numerical deconvolution of the DF80 spectrum.

To check the diamond film quality and its phase composition, Raman spectroscopy was used. During the CVD process of diamond growth, the two common phases of carbon can be produced, e.g. diamond and graphite-like phases. The Raman spectra of our set of films are presented in Figure 3.

**Figure 4:**
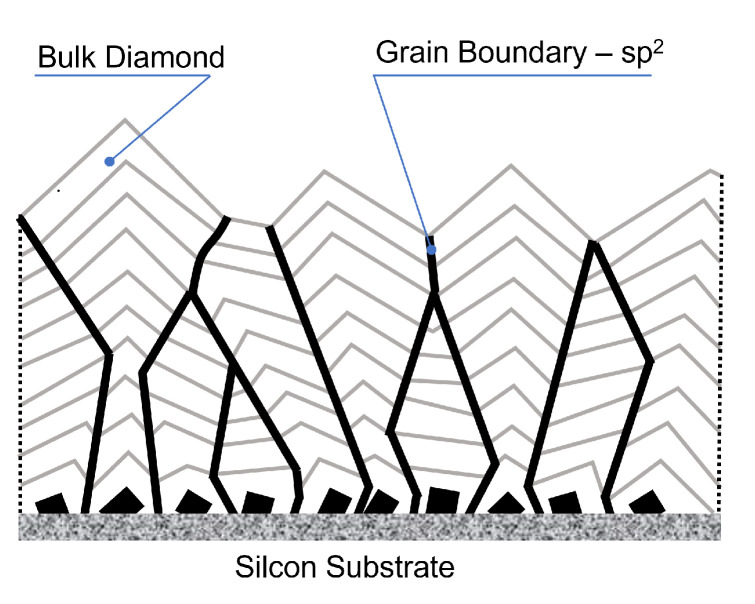
Schematic views on the structure of polycrystalline diamond films.

As it is seen, each Raman spectrum, except a sharp intense line centered at 1333 cm${}^{-1}$ ascribed to diamond structure, also shows additional broadband with a maximum at around 1530 cm${}^{-1}$ (the G-band), ascribed to sp${}^{2}$ hybridized carbon bonds characteristic to the graphite-like structure. This type of Raman spectrum clearly indicates that each of the synthesized polycrystalline diamond layers contains some amounts of amorphous carbon phase. The amorphous carbon phase with the sp${}^{2}$ hybridization occurs mainly in the form of a thin layer surrounding the microcrystallites and fills the inter-grain space as shown schematically in Figure 4.

As an indication of diamond layer quality, two parameters can be used, i.e., the FWHM of the diamond’s peak, and its phase purity described by $C_{dia}$ coefficient defined by the formula [5]:

(1) $$C_{dia}=\frac{I_{dia}}{I_{dia}+\frac{I_{G}}{50}},$$

where: $I_{dia}$—integral intensity of diamond Raman line, $I_{G}$—integral intensity of G-band in Raman spectrum. To account for the stronger resonant Raman scattering effect of sp${}^{2}$ bonded carbons, $I_{dia}$ has been scaled by a factor of 50 [32]. $I_{dia}$ and $I_{G}$ were estimated after the numerical deconvolution of each Raman spectrum [33]. The determination of the parameters is possible after numerical deconvolution of the experimental spectrum, shown for example in Figure 3b. The results of Raman spectra analysis are collected in Table 1.

Additionally, to Table 1 was introduced the concentration of hydrogen in *at*.%. It is an important parameter describing the state of the diamond surface hydrogenation. It was shown that the *H* concentration is proportional to the ratio of the slope of photoluminescence background and integral intensity of the G-band estimated from the Raman spectrum according to an empirical formula by C. Casiraghi et al. [34].

(2) $$H\left[at.\%\right]=21.7+16.6log\left(\frac{m}{I_{G}}\right),$$

where: *m*—the slope of the Raman luminescence background expressed in μm. As follows from the equation, the parameters of the spectrum of components related to the amorphous phase are used to determine the concentration of the hydrogen, i.e., the slope of the luminescence background *m* and the integral intensity of the G band. Both parameters characterized amorphous carbon admixture to diamond phase. They have been gathered in Table 2.

It should be emphasized that the degree of hydrogenation determined by the Equation (2) concerns the amorphous layer surrounding diamond microcrystallites. The model used to determine the hydrogen concentration from the luminescent background and the integral intensity of the G-band was empirically established from excitation with the 514.5 nm line from an argon laser [34]. In our case, we used 488 nm excitation. For these excitation lengths, the Raman scattering cross sections do not differ much. Casiraghi C. et al. [34] shows a linear tendency of the relationship between the background slope *m* and the integral intensity *I*${}_{G}$ of the G-band. We believe that the hydrogen concentrations can be determined by approximating the model, also taking into account the H concentration, for sure, is little lower than 20%. It must be noted that the obtained values of the hydrogen concentrations need to be treated carefully, as they display only a tendency. The FWHM of the diamond Raman line is commonly used as a diamond quality factor [35]. The FWHM for diamond monocrystal has a value around 2–2.5 cm${}^{-1}$ [36]. In the polycrystalline diamond film, the interaction of the phonons with structural defects or grain boundaries leads to the reduction of a phonon lifetime, which is inversely proportional to the FWHM of the diamond Raman line [37]. When analyzing the morphology of the obtained diamond films (Figure 1) and the results from Table 1, it can be seen that they are in excellent agreement.

### 3.4. DC-Conductivity

**Figure 5:**
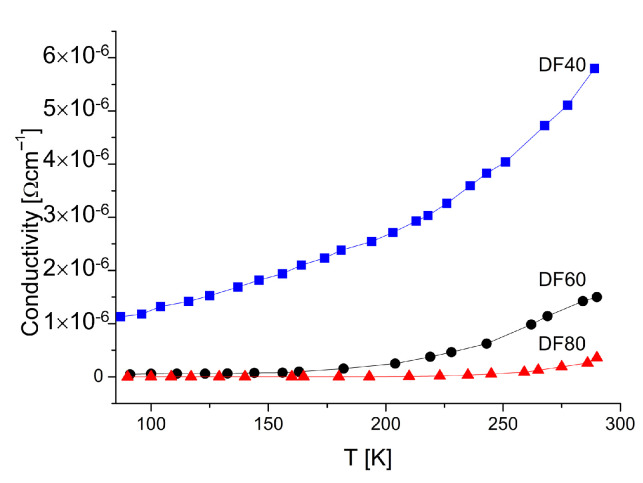
The temperature dependence of the conductivity in temperature range of 90–300 K.

To understand the electrical carrier’s transport mechanism, various contributions to carriers scattering should be taken into account. Generally, three main mechanisms could be considered, namely: (i) scattering from phonons, (ii) structural defects, dopants and (iii) grain boundaries. The last one can be the dominant factor in the case of polycrystalline materials [38]. Figure 5 shows the dependence of conductivity on temperature for three diamond layers in the temperature range from 80 to 300 K.

**Figure 6:**
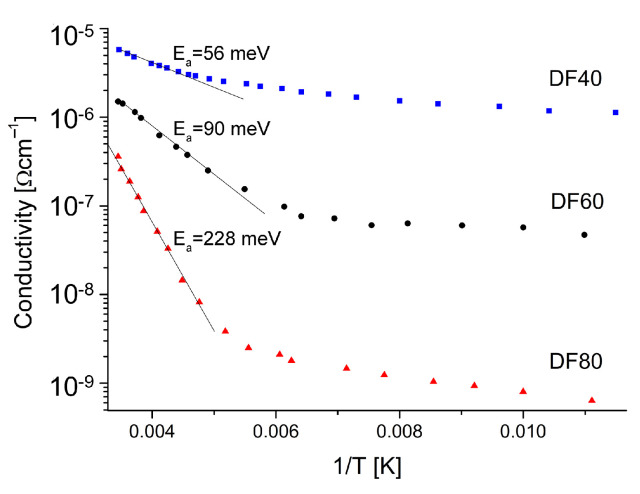
Electrical conduction variation with the inverse temperature for the studied diamond layers.

The conductivity of the films increases with temperature increase, what indicates the semiconducting behavior. The conduction properties are found to depend on the layer’s hydrogenation level (see Table 1), and the highest value possesses the DF40 sample containing up to 26 *at*.% of hydrogen. The standard plot in semilogarithmic scale, i.e., σ vs. 1/*T* Figure 6 shows two distinguishable regions that indicate different mechanisms controlling the conductivity.

In the higher temperature range of (200–300 K) the dominant mechanism is the band conduction through the extended states. The estimated activation energies, read out from the slope, see Figure 5, in this temperature range, are 56, 90 and 228 meV for the samples DF40, DF60 and DF80, respectively. It should be noted that these energies increase with the hydrogenation levels decreasing (see Table 1). It is well known that diamond is one of the best dielectric materials with the highest resistivity unless intentionally doped. One of possibilities to obtain the diamond’s semiconductor property is its surface termination by the hydrogen. It has been proposed that the hydrogen may diffuse into the diamond and contribute shallow acceptor states [39] and cause the reduction of the potential barriers’ height. This facilitated the charge carrier transport [6,40]. Taking the above into account and analysis of the morphology by SEM and the structure by XRD, one would expect that the samples characterized by bigger crystallizes without hydrogenation should exhibit higher activation energies. However, the opposite is true. Sample DF40 characterized by biggest microcrystals, the highest level of hydrogen termination shows the lowest activation energy *E*${}_{a}$. It is clear that not only structural properties as well as the hydrogen termination of the diamond surface play a key role in the increased surface conductivity.

**Figure 7:**
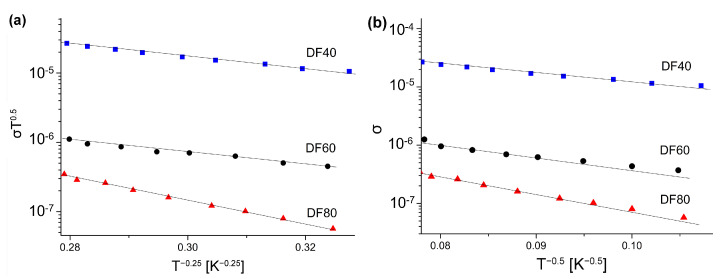
The plot of Mott’s (**a**) and Efros-Shklovskii (**b**) relations for low temperature region. Symbols are experimental data and the straight lines represent the fits.

With decreasing temperature, the conductivity of each particular layer decreases similarly as it is observed for conventional semiconductors, Figure 5. This is usually attributed to the lowering of the carrier’s concentration in the valence or conduction band for holes and electrons, respectively. However, dependencies revealed in Figure 6 show that the conventional model describing conductive properties of polycrystalline DFs can be only valid in narrow temperature range. Below specific characteristic temperature of 200 K in our case, the transport properties changes so much that the law of carrier’s concentration is no longer valid. In our opinion, one model describing whole temperature dependence is still beyond the knowledge, due to substantial difference observation of ln σ dependence versus temperature [19] instead of reciprocal temperature. Although, the low temperature carrier’s transport mechanism can be analyzed by using of the Mott Variable Range Hopping (M-VRH) if the Coulomb interactions are neglected or Efros-Shklovskii (ES-VRH) models if the long-range Coulomb interaction is taken into account [41,42,43]. In the M-VRH model, the conduction is described by the formula:

(3) $$\sigma T^{0.5}=\sigma_{00}exp{\left(-\frac{B}{T}\right)}^{0.25},$$

and in the ES-VHR model:

(4) $$\sigma =\sigma_{0}exp{\left(-\frac{T_{ES}}{T}\right)}^{0.5},$$

where: $\sigma_{00},B,\sigma_{0},T_{ES}$—are constants. The corresponding values of these constants can be evaluated from the linear slopes of the corresponding plots presented in Figure 7a,b for the M-VHR and the ES-VRH models, respectively. They both show a good linear relationship. It is difficult, therefore, to determine which of the hopping mechanisms is observed. Such a problem is commonly perceived for other materials and requires additional analysis [44]. In our case, we decided to use the M-VRH model due to the higher correlation coefficients 0.998 instead of 0.987 achieved for this model. It seems reasonable to assume that the diamond’s surface is compensated, and long-range Coulomb interaction can be neglected. The density of states, *N*(*E*${}_{F}$) at the Fermi energy is inversely proportional to the *B* parameter in Equation (3) [45]:

(5) $$B^{4}=T_{M}=18.1\frac{\alpha^{3}}{kN(E_{F})},$$

where: $T_{M}$—is the characteristic of Mott’s temperature, *k*—Boltzmann’s constant, α—describes the localization of the wave function. The parameter α can be estimated using the following formula [46]:

(6) $$\alpha =22.52\sigma_{00}T_{M}^{0.5},$$

The average hopping distance (*R*) and hopping energy (*W*), which depend on the density of states, are given by formulas [47].

(7) $$R={\left[\frac{9}{8\pi \alpha kTN(E_{F})}\right]}^{\frac{1}{4}},W=\frac{3}{4\pi R^{3}N\left(E_{F}\right)}.$$

The calculated values of *N*(*E*${}_{F}$), *R* and *W* according to the M-VRH model are listed in Table 3.

As it can be seen from the table, with the increase in the hydrogenation level and the crystallite size (see Table 1), the mean hopping distance R increases, while other parameters decrease. On the one hand, the hydrogen can passivate of certain defects (for example dangling bonds) resulting in lower charge’s concentration. However, on the other side, the better quality of the crystal structure gives an opportunity for the depletion layer creation [10]. In the mechanism of charge transfer doping, the surface’s hydrogen function as the acceptor [39,48]. In this way, a change of electrostatic equilibrium on one side of the grain can be easily propagated to another one. This explains the tendency being seen between parameters presented in the Table 3. The grain boundary can be defined as the region between two grains where crystal orientation changes. In the interface between grains, the atoms are disordered. It is guiding to the creation of a high concentration of defects and hydrogen atoms as well. They can act as trapping centers and reduce the number of free carriers available for electrical transport phenomenon. After carriers capturing the trap centers become electrically charged, and potential energy barriers are formed, reducing carriers’ mobility between grains. Assuming that the grains are partially depopulated, the electrical conductivity through the grain boundaries can be described by the formula [16]:

(8) $$\sigma T^{0.5}=Aexp\left(-\frac{e\phi_{GB}}{kT}\right),$$

where: *e*—electron charge, *A*—constant. The value of the $\phi_{GB}$ can be estimated from the linear part of the slope of ln($\sigma T^{0.5}$) vs. 1/*T*. The estimated $\phi_{GB}$ are summarized in Table 3. The obtained values of the potential barriers confirm the fact of defects passivation on the grain boundaries, for example dangling bonds, acting as trapping centers of the charge carriers.

## 4. Conclusions

We presented an analysis of the transport properties of un-doped diamond films grown at different gas pressure. Samples characterized by varying levels of hydrogen termination were synthesized for this purpose. The dc-electrical studies of the obtained diamond films reveal that the transport properties can be known by extrapolation in narrow temperature range where the carrier’s concentration law is valid. In our case, at temperature lower than 200 K the M-VHR model can be successfully utilized. As at higher temperatures, the principal conduction mechanism is the thermally activated one. It has been shown that the surface hydrogenation plays the crucial role in tuning transport properties. With its rice, there is observed increase in the surface conductivity, decrease in the grain boundaries barrier $\phi_{GB}$ whereas the density of the localized states near the Fermi level *N*(*E*${}_{F}$) was found to decrease from 4.7$\;\times \;10^{14}$ eV${}^{-1}$ cm${}^{-3}$ to 9.2$\;\times \;10^{13}$ eV${}^{-1}$ cm${}^{-3}$. When the average hopping energy *W* increases from 32 meV to 57 meV, with the average hopping distance *R* decreases from 4.2$\;\times \;10^{-4}$ cm to 2.2$\;\times \;10^{-5}$ cm. In our opinion, the presented results indicate that the degree of hydrogenation may significantly impact the electrical properties of diamond layers.

## Figures and Tables

**Table 1:** The structural properties of diamond films derived from Raman spectra and XRD.

Films	Pressure	Peak Position	FWHM	*C* ${}_{\mathrm{dia}}$	*H*	*L*
	[hPa]	[cm${}^{-1}$]	[cm${}^{-1}$]	[a. u.]	[*at*.%]	[nm]
DF40	40	1331.9	6.8	0.99 ± 0.01	26 ± 1	66 ± 1
DF60	60	1331.7	7.9	0.98 ± 0.01	24 ± 1	52 ± 1
DF80	80	1331.6	9.2	0.96 ± 0.01	17 ± 1	35 ± 1

**Table 2:** The G-band parameters and photoluminescence slope.

Films	Peak Position	FWHM	Integral Intensity	*m*-Slope
	[cm${}^{-1}$]	[cm${}^{-1}$]	[a. u.]	μm
DF40	1531	243	53,125	103,000
DF60	1534	185	45,180	64,000
DF80	1534	125	25,030	13,000

**Table 3:** The calculated parameters of the M-VRH model.

Films	*N*(*E*${}_{F}$)	*R*	*W*	$\phi_{\mathrm{GB}}$
	[cm${}^{-3}$]	[cm]	[meV]	[meV]
DF40	9.2 $\times \;10^{13}$	4.2$\;\times \;10^{-4}$	32	60
DF60	2.6$\;\times \;10^{14}$	2.5$\;\times \;10^{-5}$	33	110
DF80	4.7$\;\times \;10^{14}$	1.23$\;\times \;10^{-5}$	57	257

## Abbreviations

The following abbreviations are used in this manuscript:

HF CVD	hot filament chemical vapor deposition
M-VRH	Mott variable range hopping
ES-VRH	Efrost-Shklovskii variable range hopping
I-V-T	current voltage characteristics versus temperature
RT	room temperature
GB	grain boundary
*N*(*E*${}_{F}$)	density of states near Fermi energy
$\phi_{GB}$	grain boundary’s potential
DF	diamond film
SEM	scanning electron microscopy
FWHW	full width at half maximum
